# Enhanced Recovery After Surgery (ERAS) Protocols in Pancreatic Cancer Resections: Their Impact on Postoperative Morbidity

**DOI:** 10.7759/cureus.97007

**Published:** 2025-11-16

**Authors:** G Harsha Vardhan Reddy, Manu Pandya, Hement Kumar Ahirwar, Shipra Saklani, Sukanta Bandyopadhyay

**Affiliations:** 1 Department of Hepato-Pancreato-Biliary (HPB) and Liver Transplantation, Institute of Liver and Biliary Sciences, New Delhi, IND; 2 Department of Clinical Medicine, Saket Nursing Home, Jabalpur, IND; 3 Department of General Surgery, Chhindwara Institute of Medical Sciences, Chhindwara, IND; 4 Department of Food Technology and Nutrition, Lovely Professional University, Phagwara, IND; 5 Department of Biochemistry, Rama Medical College Hospital and Research Centre, Kanpur, IND

**Keywords:** enhanced recovery after surgery, enhanced recovery protocols, pancreatic cancer, perioperative care, postoperative complications

## Abstract

The sole treatment for pancreatic cancer, which is a hazardous condition, is surgery, which has a high postoperative morbidity rate. Traditional perioperative care often makes the healing process more difficult and increases the risk of problems. The Enhanced Recovery After Surgery (ERAS) protocols that were created to address colorectal operations are intended to alleviate surgical trauma and promote recovery based on evidence-based, multimodal interventions. The current review will focus on the ongoing problem of the high rate of pancreatic resection morbidity and consider the effectiveness and applicability of ERAS in the same context. The aim is to critically evaluate clinical outcomes and barriers to implementation by performing a narrative synthesis of trials, observational studies, and meta-analyses. The important results imply that the use of ERAS protocols minimizes complications, decreases the length of hospital stays, and enhances recovery without mortality or readmission. Key challenges are the emphasis on protocol compliance, institutional issues, and limited representation of high-risk groups, namely, the elderly and infirm. The review concludes that, although ERAS has apparent advantages, its success is based on readiness, a robust multidisciplinary approach involving surgeons, anesthesiologists, nurses, dietitians, physiotherapists, and pain specialists to ensure coordinated care, and high protocol fidelity of the institution. The implications are that ERAS guidelines will have to be standardized, digital adherence tools will also be necessary, and multicenter trials will need to be inclusive of diverse patient populations. The review offers an elaborate model that can be used to achieve improved, fairer, and patient-centered perioperative care in pancreatic oncology.

## Introduction and background

Pancreatic cancer belongs to the number of the deadliest cancers worldwide, and its prognosis after five years of survival is less than 10% even with the development of diagnostic and treatment methods [1]. By the year 2023, pancreatic cancer is estimated to cause about 466,000 deaths per annum, and by the year 2033, it will be the second most frequent cause of death due to cancer in several high-income nations [2]. This has been attributed mostly to aggressive tumor biology, absence of early symptoms, and limited treatment choices at the time of diagnosis, which have contributed to the poor prognosis. When first presented, only 15-20% of patients can be considered candidates for curative resection since most of them are presented at an advanced or metastatic stage [3].

Surgical excision is the only treatment for pancreatic cancer that may be curative, but it is a procedure that carries a lot of morbidity. The most common surgical methods are pancreatoduodenectomy (Whipple procedure), distal pancreatectomy, and, less often, total pancreatectomy. These operations are technically challenging since the pancreas is retroperitoneal, and it is near vital vascular structures such as the superior mesenteric veins and portal vein [4]. Nevertheless, even with the impressive developments in surgical methods, postoperative care, and morbidity after pancreatic resections have not been reduced to below 30-60% even in high-volume centers [5]. Complications such as delayed gastric emptying, postoperative pancreatic fistula, intra-abdominal collections, and sepsis are common and may extend the hospital stay, increase expenses, postpone the administration of adjuvant treatments, and hence adversely affect long-term outcomes [6].

The first Enhanced Recovery After Surgery (ERAS) protocols were developed by Kehlet in the late 1990s in colorectal surgery, and ERAS is now a multimodal, multidisciplinary approach that aims to minimize the physiological cost of surgery to aid a quicker recovery to the preoperative state [7]. To improve patient outcomes, ERAS, a multi-modal evidence-based perioperative bundle, combines numerous evidence-based perioperative components into a structured care route [8]. These protocols have been accepted in colorectal surgery with decreased rates of complications, decreased length of hospital stays, and patient satisfaction and are now being investigated in hepatobiliary and pancreatic surgeries [9].

**Figure 1:**
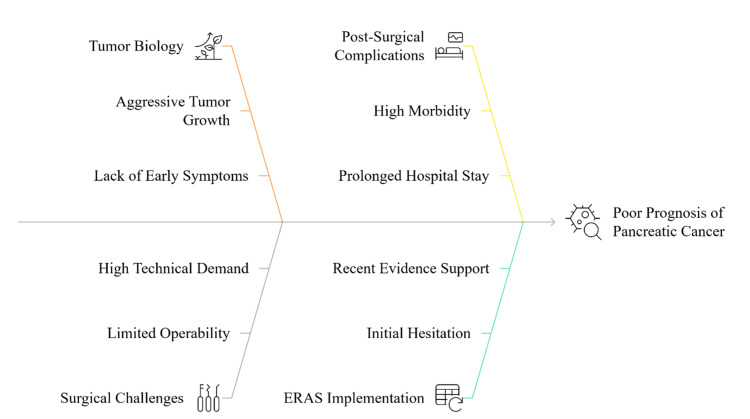
Key contributing factors to the poor prognosis of pancreatic cancer Created by the authors

In pancreatic surgery, ERAS has traditionally been conservative because of the fears of the feasibility and safety of early postoperative interventions on a high-risk patient cohort, including feeding and drain removal. However, over the last decade, there has been an ever-growing body of evidence indicating that, when properly selected and protocol adjusted to individual patients, ERAS may be safe and even helpful in pancreatic resections. A number of retrospective and prospective studies have shown that there were decreases in the length of stay, post-operative complications, and medical expenses without an increase in the readmission or mortality rates [10]. Figure 1 illustrates the key factors contributing to the poor prognosis of pancreatic cancer, including aggressive tumor biology, surgical complexity, postoperative complications, and the evolving role of ERAS protocols.

Objectives of the review

This review aims at the critical evaluation of the available literature on the implementation and the results of ERAS protocols in patients who undergo pancreatic resection with cancer. In particular, the review was conducted to evaluate the effect of ERAS on postoperative morbidity, consider separate elements and adherence rates of ERAS pathways when performing pancreatic surgery, and determine the challenges and limitations of their implementation. This article is also aimed at identifying the gaps where additional research is necessary and offering suggestions that can be used to guide clinical practice and guideline development in the future by synthesizing the information presented in clinical trials, observational studies, and meta-analyses.

## Review

Principles and components of ERAS

The systematic, evidence-based perioperative treatment known as "Enhanced Recovery After Surgery" (ERAS) approach is designed to optimize outcomes by reducing surgical stress. Originally developed for colorectal procedures, ERAS integrates multimodal interventions across surgical, anesthetic, nutritional, and rehabilitative domains. As experience and evidence have expanded, these protocols are increasingly being applied to pancreatic resections, with growing support for their ability to reduce complications, accelerate recovery, and restore function more efficiently [11]. A cornerstone of ERAS is patient-centered care, particularly through preoperative engagement. Patient education is paramount, as patients need to be fully onboard and understand the purpose and importance of each perioperative step, including postoperative rehabilitation, to ensure compliance. Educating patients about the surgical process and recovery expectations empowers them to participate actively in their care, resulting in lower anxiety and higher adherence [12]. This emphasis on engagement and preparation is especially important in pancreatic surgery, where patients often present with cachexia, cholestasis, and overall poor nutritional status. Optimizing nutrition before surgery strengthens immune function and may improve healing outcomes [13].

ERAS also challenges traditional practices, such as prolonged preoperative fasting. Allowing clear, carbohydrate-rich fluids up to two hours before anesthesia helps maintain metabolic balance and preserves lean body mass, without compromising safety [14]. On the intraoperative front, utilization of goal-directed hydration treatment with opioid-sparing analgesia reflects the shift toward minimizing side effects such as nausea, ileus, or fluid overload, especially relevant in major abdominal surgeries [15,16]. The postoperative phase of ERAS emphasizes early recovery milestones that were once considered controversial in pancreatic surgery, such as early mobilization and oral intake. Recent findings support the safe application of these strategies in selected patients, showing trends toward reduced delayed gastric emptying and improved gastrointestinal function [17]. Similarly, while minimally invasive techniques align well with ERAS goals, their use in complex operations such as pancreatoduodenectomy remains limited to high-volume, specialized centers [18].

Some elements of ERAS remain subjects of debate and require personalized approaches, most notably, the use of intra-abdominal drains. While removal of the drain early is encouraged in low-risk patients, those with soft glands or small ducts still require individualized assessment due to their high risk for postoperative pancreatic fistula (POPF) [19]. Ultimately, ERAS's success is linked to adherence. Strong institutional support, continuous training, and performance monitoring systems are crucial to achieving consistent protocol application. Studies consistently show that higher adherence correlates with improved clinical outcomes, reduced hospital stays, and a faster return to normal functioning [20]. Table 1 complements this overview by outlining the specific components of ERAS protocols across each perioperative stage, along with their intended benefits and supporting references.

**Table 1:** ERAS components and goals in pancreatic surgery ERAS: Enhanced Recovery After Surgery

Phase	ERAS Component	Purpose/Outcome	Notes/Considerations
Preoperative	Preoperative education	Increases patient engagement, reduces anxiety, and improves adherence [12]	Sets clear expectations for recovery
	Nutritional optimization	Improves immune function and wound healing [3]	Oral immunonutrition with arginine, omega-3s; screen early
	Reduced fasting	Maintains insulin sensitivity, lean mass [14]	Clear fluids allowed up to 2h, solids up to 6h before anesthesia
Intraoperative	Opioid-sparing analgesia (e.g., epidural, TAP block)	Reduces ileus, nausea, and sedation [1]	Multimodal pain control approach
	Goal-directed fluid therapy (GDFT)	Maintains normovolemia; prevents overload and hypoperfusion [16]	Personalized fluid strategy under study in pancreatic surgery
Postoperative	Early mobilization (within 24h)	Reduces thromboembolism, enhances pulmonary and bowel function [17]	Standard component of ERAS
	Early oral intake	Reduces delayed gastric emptying (DGE), supports GI function [7]	Safe in well-selected patients; requires stratification
	Minimally invasive surgery (e.g., laparoscopic DP)	Decreases surgical trauma, shortens recovery [18]	Best for distal pancreatectomy; PD is limited to specialized centers
	Drain management	Reduces POPF risk through selective use and early removal [19]	Personalized, high-risk patients may still require drains
Cross-cutting	Protocol adherence	Decreased complications, LOS, and functional recovery [10]	Needs institutional support, training, and performance audits

Historical context: ERAS in pancreatic vs. colorectal surgery

In the 1990s, Kehlet presented the ERAS scheme in the field of colorectal surgery [5]. This was demonstrated to be true early on since the reduction of metabolic response to surgical trauma was found to have a significant reduction in length of stay in the hospital and the complication rates without readmission or death being a new standard of perioperative care [21]. Within a decade, the ERAS protocols became common in colorectal surgery and were considered a standard in other surgical fields [8]. Nevertheless, the implementation of ERAS in pancreatic surgery started slowly at the beginning [6]. Parenchymal resections of the pancreas, in particular, pancreatoduodenectomy (Whipple procedure), are technically complex and are associated with high rates of morbidity on their own [10]. The clinical unwillingness to use ERAS in such a setting was due to early oral feeding, fluid restriction, and lack of prophylactic drains [12].

The landmark was the publication of pancreas-specific ERAS recommendations by the ERAS Society in 2012 [7]. Even though some of the main principles, such as preoperative education, opioid-sparing analgesia, and early mobilization, could be easily used in pancreatic resections by colorectal models, there were some of them that had to be modified due to the specifics of the surgery [13]. To give an example, the integrity of anastomotic sites and a high degree of variation in the technique of reconstruction resulted in more conservative use of early enteral nutrition and drain management [15]. Over the years, the observational cohort and prospective trial evidence suggested that ERAS is safe to be applied in pancreatic surgical practice, particularly at institutions with a large number of patients and experienced multidisciplinary teams [9]. It has been reported to have results of shorter hospital stay and decreased infections, and the earlier start of adjuvant therapy [14]. Nevertheless, experience, infrastructure, and staff training of an institution play a gigantic role in determining the success of ERAS, and it is not equally applied all over the world [22]. The fact that, having appeared in colorectal surgery, ERAS was transferred to pancreatic surgery also proves that its ideas are universal and adaptable [11]. Even though some points had to be corrected, the very idea of mitigating the surgical stress and normalizing the recovery is feasible and better justified in the treatment of pancreatic cancer [5].

Perioperative Risk Profile in Pancreatic Cancer Resection

Surgery difficulty and patient-related risk of malnutrition, cholestasis, diabetes, and immunosuppression make the profile of perioperative risk of resection of pancreatic cancer particularly elevated [3]. In well-experienced centers, the morbidity rates following pancreatoduodenectomy are not less than 40% at best [11]. The emergence of complications such as POPF, delayed gastric emptying (DGE), hemorrhage, and intra-abdominal abscesses influences the recovery process and the period of adjuvant therapy in a very important way [7]. POPF is clinically the most significant complication and is a result of leakage at the pancreatic anastomosis [2]. Depending on its severity, it may lead to sepsis, delayed healing, and even reoperation [10]. The rate is 10-30%, and it is related to the gland texture, the size of the duct, and the operative technique [1]. DGE is also defined by the insensitivity to oral feeding following the seventh day of the operation [13]. It appears in a third of the patients and is one of the frequent causes of prolonged stay in the hospital [8].

These risks target the ERAS strategies through the application of multimodal and preemptive interventions [9]. Optimization of nutrition before the surgery and smoking cessation improves wound healing and reduces the number of infections [4]. In the course of surgery, GDFT reduces edema of the bowel and anastomotic tension [15]. The regional analgesia is free of ileus and respiratory issues and does not cause unnecessary sedation [5]. ERAS postoperative principles aim at early but cautiously titrated oral nutrition that is typically initiated with liquid foods and advanced as tolerated [14]. Even though the early feeding is evidence-based, it is important to state that this practice is debatable in the case of a high risk of POPF or DGE [16]. Clinical monitoring and protocol flexibility are essential [12]. Similarly, personalized choices in regards to drain utilisation and clearance are also guided by the utilization of selective drain use by the intraoperative scoring systems, e.g., fistula risk score [18].

**Figure 2:**
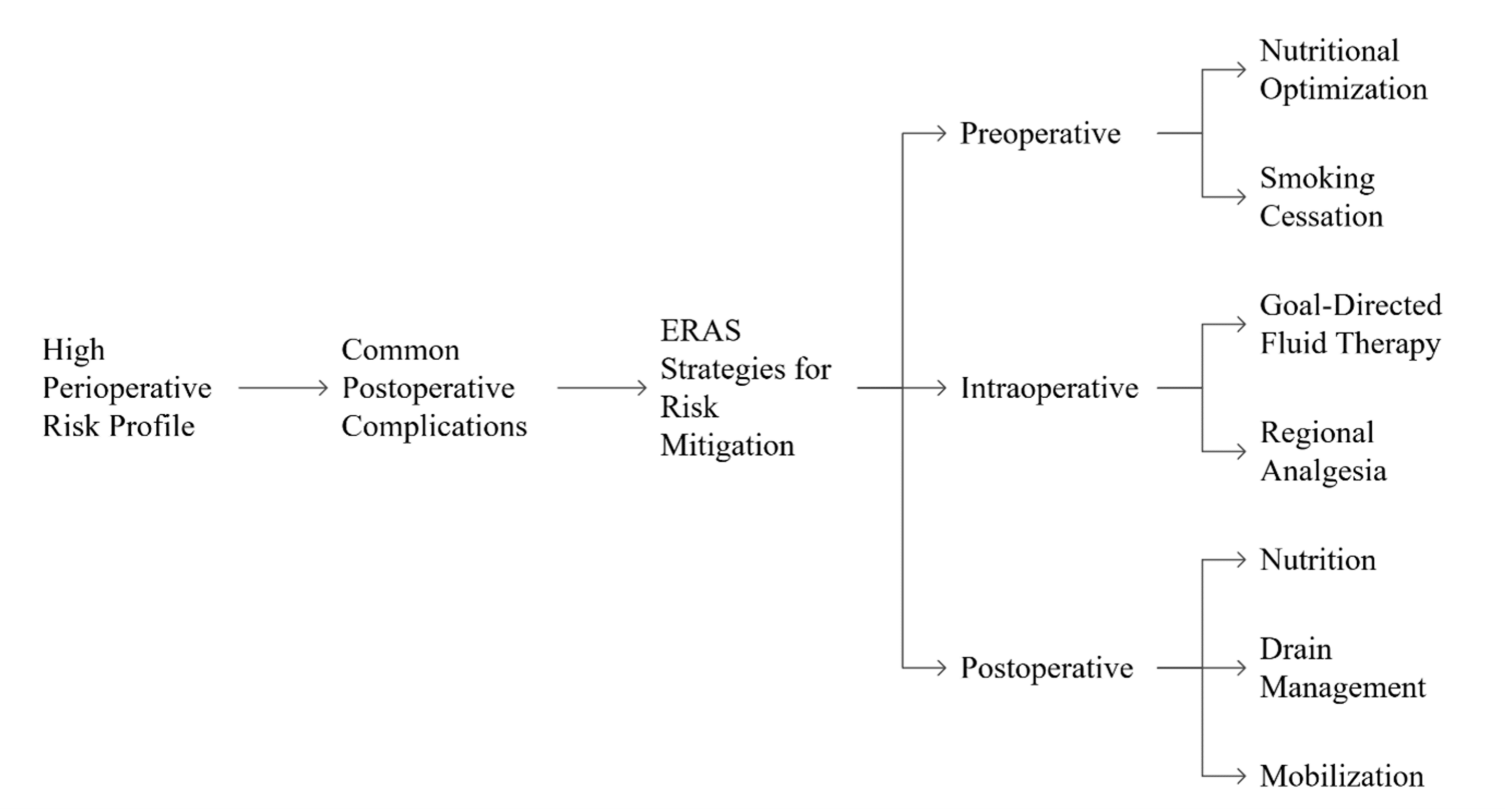
ERAS-based risk mitigation strategies in pancreatic surgery Created by the authors

Multimodal, opioid-sparing analgesia can early mobilize patients and, therefore, reduce pulmonary infections and thromboembolic events [6]. These measures in combination constitute a synergistic risk mitigation framework [17]. It is, however, worth noticing that ERAS does not prevent complications, but rather it changes the severity and the frequency of complications in case the protocol is followed strictly [9]. The meta-analyses have continually shown that pancreatic ERAS pathways are associated with reduced overall morbidity, reduced time to functional recovery, and do not augment readmissions or mortality when compared to conventional care [22]. These benefits are most obvious in settings where compliance is high, and this is the reason why institutional protocols, audit systems, and patient selection criteria are highly treasured [10]. Figure 2 illustrates a chain of events, starting with a high-risk profile of pancreatic cancer surgery, typical postoperative complications, and their ERAS interventions that can be utilized to prevent the risks.

Preoperative ERAS Elements

The key to ERAS is effective preoperative optimization, especially in the surgery of pancreatic cancer patients who usually come with poor nutritional and functional status. The most important of them are nutritional screening, behavioral modification, prehabilitation, and patient education. More than half of the pancreatic cancer patients are affected by malnutrition, which has been associated with high morbidity and slow recovery [10]. ERAS suggests the regular screening with validated instruments, such as NRS-2002 or the Subjective Global Assessment, to detect nutritional risk [23]. When present, nutritional intervention should begin promptly. Randomized trials have shown that immunonutrition, arginine-enriched, omega-3 fatty acids, and nucleotide-enriched formulas have an effect of reducing infectious complications and shortening hospital stay [24].

Behavioral risk factors also require targeted intervention. Smoking and alcohol use independently increase postoperative complications [25]. Smoking affects the functioning of the lungs and the healing of anastomosis, and alcohol affects the immune system and the functioning of the liver [26]. Cessation of at least four weeks before surgery has been found to decrease the complication rates and is to be encouraged [27]. Prehabilitation is usually two to four weeks of physical training, nutritional support, respiratory exercises, and psychological preparation. These interventions are beneficial to postoperative independence, rates of complications, and time to baseline function [28]. Patient education is essential. The structured counseling will enhance the level of postoperative goals completion (early mobilization and oral intake), anxiety, and satisfaction [29]. Early patient involvement promotes shared decision-making and prepares patients to play an active role in their recovery. The combination of these preoperative interventions enhances the resilience of patients and preconditions a better postoperative process [30].

Intraoperative ERAS Strategies

The intraoperative phase of ERAS plays a pivotal role in shaping postoperative outcomes by aiming to preserve physiological homeostasis and minimize surgical trauma. This is achieved through a carefully coordinated set of strategies that go beyond traditional surgical protocols. While Table 2 outlines these specific measures, this section provides insight into how and why they contribute to improved recovery in pancreatic surgery. One of the central intraoperative principles is personalized fluid management, which shifts away from fixed-volume infusions to real-time, data-guided therapy. Goal-directed fluid therapy (GDFT) relies on physiological markers such as stroke volume variation and cardiac output to fine-tune volume resuscitation. Although widely supported in abdominal surgery, more excellent research is required to confirm its full benefits in pancreatic procedures [22,31].

**Table 2:** Intraoperative ERAS measures and their clinical roles in pancreatic surgery ERAS: Enhanced Recovery After Surgery

Strategy	Purpose	Key Considerations
Goal-Directed Fluid Therapy (GDFT)	Maintains normovolemia; prevents fluid overload and hypoperfusion [22]	Uses dynamic variables (e.g., stroke volume, cardiac output); limited pancreatic-specific evidence
Minimally Invasive Surgery (MIS)	Reduces tissue trauma, postoperative pain, and hospital stay [32]	Best evidence for laparoscopic distal pancreatectomy; robotic techniques limited to high-volume centers
Patient Selection for MIS	Ensures the safety and feasibility of MIS in pancreatic cases [2]	Tumor size, vascular involvement, and obesity may limit MIS use
Multimodal Analgesia	Minimizes opioid use; supports early mobilization and gut function [13]	Includes acetaminophen, NSAIDs, and regional anesthesia (TAP block, epidurals)
Anti-inflammatory Adjuncts	Reduces postoperative inflammation; enhances early recovery [33]	Includes dexamethasone, lidocaine, and the use of short-acting agents
Normothermia Maintenance	Prevents hypothermia-related complications (bleeding, infection, delayed discharge) [7]	Active warming devices and warmed IV fluids maintain core temperature above 36°C

The growing emphasis on minimally invasive techniques also aligns well with ERAS objectives. Laparoscopic distal pancreatectomy, in particular, has demonstrated reduced tissue trauma, lower postoperative pain, and shorter hospital stays. However, the technical complexity of more extensive procedures such as pancreatoduodenectomy means that such approaches remain largely restricted to high-volume, specialized centers. Appropriate patient selection, based on tumor size, vascular involvement, and body habitus, is critical to the safe and effective application of minimally invasive surgery (MIS) [32]. Anesthetic protocols under ERAS stress the importance of multimodal analgesia in reducing opioid dependence. This not only helps avoid opioid-related complications such as ileus and nausea but also facilitates early mobilization and feeding. Agents such as acetaminophen and NSAIDs are typically used alongside regional techniques such as epidurals or TAP blocks. Additionally, the use of intraoperative adjuncts such as dexamethasone and lidocaine has been associated with better inflammatory control and smoother recovery trajectories [33].

Maintaining normothermia during surgery is another essential but often underestimated target. Hypothermia has been linked to adverse outcomes, including increased blood loss and infection risk. To counter this, ERAS protocols include active warming systems and the use of warmed IV fluids to keep the patient’s core temperature above 36°C [34]. Together, these intraoperative strategies form a comprehensive approach that supports the overall ERAS goal: reducing the physiological burden of surgery and paving the way for earlier mobilization, faster gastrointestinal recovery, and ultimately, shorter hospital stays. Table 2 summarizes these interventions, their clinical aims, and supporting evidence.

Postoperative ERAS Protocols

Postoperative ERAS interventions are aimed at the early recovery process, the avoidance of complications, and the safe discharge. Key domains include pain management, nutrition, and mobilization. Pain control relies on opioid-sparing multimodal analgesia. NSAIDs and acetaminophen are regularly employed, and regional blocks are to be used, such as TAP or thoracic epidurals. Although the latter are effective in open surgery, they are progressively used selectively because of the hazards of hypotension and urinary retention [35]. The proper management of pain allows earlier ambulation, enhances respiratory activity, and decreases ileus.

Early enteral nutrition is promoted 24-48 hours after the surgery, assuming that the patient can accept oral feedings. Early feeding maintains gut barrier, enhances immunity, and decreases catabolism. This method has been proven to be safe and possibly lowers the infection and restores bowel functions [36]. Diet is typically advanced stepwise from liquids to solids. When oral intake is not timely, enteral nutrition through a nasojejunal tube is recommended as opposed to parenteral nutrition because it has fewer risks of complications. One of the best indicators of recovery is early mobilization. The patients are motivated to walk the first day after the surgery and become more active every day [37]. This minimizes the chances of pulmonary complications, deep vein thrombosis, and muscle wasting. Unnecessary lines, drains, and catheters should be eliminated early to help in mobilization.

Discharge is now determined by the successful attainment of functional milestones: the successful control of pain with oral analgesics, the ability to tolerate a regular diet, the ability to ambulate independently, and the willingness of the patient to leave [32]. These criteria improve discharge timing and reduce readmissions. ERAS programs have monitoring of compliance as a routine part of the programs to monitor protocol adherence and outcomes. Standardized order sets, nursing checklists, and electronic dashboards are tools to enable support of adherence [21]. Better adherence to postoperative factors has been linked independently to fewer complications, reduced length of stay, and increased patient satisfaction. Overall, postoperative ERAS management in pancreatic surgery assists in the transition of care towards active recovery through quantitative goals, which maximize outcomes and minimize resource consumption.

Clinical outcomes of ERAS in pancreatic surgery

ERAS protocols have been shown to show a significant positive change in postoperative outcomes after pancreatic resections. The second most unanimous result in the literature is a decrease in length of stay (LOS) in the hospital, with the majority of studies recording a mean decrease of two to four days when compared with traditional care pathways [38]. Such results are enabled by previous ambulation, fewer complication rates, and standardized discharge milestones [1]. Other postoperative problems and ERAS have also been used to decrease surgical site infections (SSIs). Premature enteral nourishment, multimodal analgesia, and normothermia, in combination, stimulate immune response and decrease gastrointestinal stasis, which lowers the risk of infection [15]. As an illustration, the multicenter prospective study revealed that total complication rates were cut in half (from 50% to 35%) once ERAS was used [39].

POPF is another complication that has not been eliminated; however, ERAS pathways were linked to decreasing the severity of clinically relevant Grade B/C fistulas [40]. It is assumed that this advantage can be attributed to a decrease in systemic inflammation and promotion of anastomotic healing because of the improvements in nutrition and fluid management [9]. Although early discharge may imply the possibility of high readmission rates, there is no evidence to prove this premise. The available literature shows similar 30-day readmission rates in ERAS and conventional care, and the rates related to the ERAS usually vary between 12% and 16% [41]. To some degree, even the evidence indicates that late readmissions can be decreased by enhanced early recovery and uniform follow-up [3].

**Figure 3:**
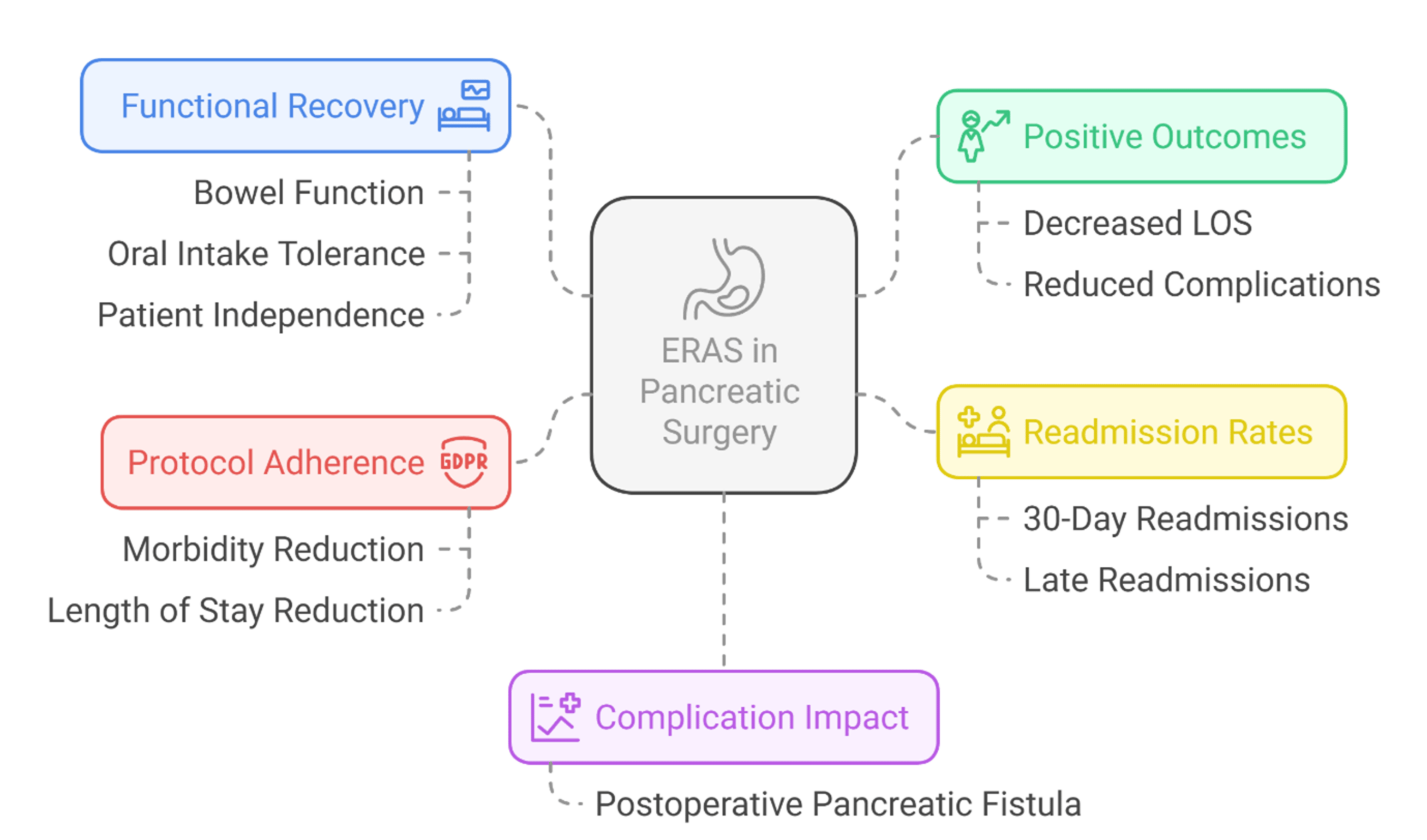
Important effects of ERAS procedures in pancreatic surgery Created by the authors ERAS: Enhanced Recovery After Surgery

The outcomes of functional recovery, including the time of bowel movement, the ability to tolerate oral intake, and the independence of the patient, are also better under ERAS [22]. These benefits lead to greater patient satisfaction of patients and quicker recovery of preoperative functionality [42]. Importantly, the magnitude of ERAS benefit correlates with adherence. Researchers found that, if more than 70% of the protocol components were adhered to, morbidity was reduced significantly, and LOS decreased [43]. Thus, protocol fidelity is one of the determinants of ERAS effectiveness in pancreatic surgery. Although the ERAS protocols have enhanced the quality of care, implementation of these measures is only successful through evidence-based design, but more importantly, consistent application, which is discussed in the next section. Figure 3 shows that ERAS protocols enhance recovery in pancreatic surgery by improving outcomes and reducing complications without increasing readmissions.

Compliance and implementation barriers

Although the clinical advantages of pancreatic surgery with ERAS are increasingly well-documented, the extent to which these benefits are realized in practice largely depends on achieving high compliance with protocol components and ensuring cohesive multidisciplinary implementation. Studies suggest that adherence to at least 70% of ERAS elements is necessary to observe meaningful improvements in outcomes [44]. However, adherence rates vary widely among institutions due to both systemic and human factors. At the institutional level, structural limitations such as the lack of ERAS coordinators, fragmented leadership, and poor interdepartmental communication have been identified as major barriers to effective implementation [7]. In many settings, key components such as early mobilization or nutritional initiation are inconsistently applied due to the absence of clearly assigned responsibilities [4]. Furthermore, without robust auditing and feedback mechanisms, protocol deviations often go untracked, diminishing the ability to evaluate and refine practices [12].

Surgeon-related challenges also play a critical role. Some practitioners remain hesitant to adopt core ERAS elements, including early feeding and conservative fluid strategies, particularly when managing high-risk or complex cases [8]. This resistance is often rooted in concerns about disrupting established routines or a lack of familiarity with ERAS processes. These concerns are compounded in low-volume centers, where teams may have limited exposure to or experience with ERAS implementation [45]. From the patient's perspective, several factors may limit protocol adherence. Frailty, cognitive impairment, comorbidities, and the absence of caregiver support can interfere with early ambulation and nutritional goals [23]. While individualized care modifications are often necessary, they can lower overall adherence rates and complicate outcome monitoring [20]. Moreover, protocol variability across institutions, such as differences in nasogastric tube removal timing or mobilization schedules, poses a challenge to the standardization and benchmarking of ERAS practices. Even with formal guidelines from the ERAS Society, local adaptations remain common and make cross-institutional comparison and replication of best practices more difficult [6,13].

One of the most underdeveloped yet essential elements is staff education. Institutions that invest in interactive training, interdisciplinary workshops, and simulation-based learning report better coordination, improved protocol adherence, and enhanced patient outcomes [4,21]. Successful ERAS programs also leverage technology-enabled tracking tools such as electronic dashboards, standardized order sets, and nursing checklists to monitor compliance in real time, identify performance gaps, and support continuous quality improvement [11]. Ultimately, overcoming these implementation barriers requires a combination of institutional leadership, data-driven evaluation, and a culture grounded in evidence-based, collaborative care. Without these foundational supports, the proven benefits of ERAS risk are being inconsistently applied or diluted in practice. Table 3 complements this discussion by summarizing specific barriers and corresponding strategies for effective ERAS execution across institutional, clinical, and patient domains.

**Table 3:** Barriers and solutions for effective ERAS implementation in pancreatic surgery ERAS: Enhanced Recovery After Surgery

Category	Barriers	Solutions
Institutional	Lack of ERAS coordinator, weak interdepartmental communication, and absence of audits/feedback loops [12]	Appoint ERAS leadership, promote team-based communication, and implement audit & feedback systems.
Protocol Compliance	Task ambiguity (e.g., who manages nutrition/mobilization) and inconsistent protocol application [4]	Assign responsibility clearly and standardize ERAS components across teams
Surgeon-Level	Reluctance toward early feeding & fluid limits and fear of disrupting routine [8]	Evidence-based education and involvement in protocol development
Center Volume	Low-volume centers lack ERAS exposure or experience [44]	Cross-institutional mentorship and knowledge transfer from high-volume centers
Patient-Level	Frailty, comorbidities, cognitive and psychosocial limitations, and limited family support [23]	Individualized care plans and integration of support services (social work, caregivers)
Protocol Variation	Local differences (e.g., drain removal timing, mobilization) hinder standardization [13]	Follow ERAS Society guidelines and justify/document local adaptations
Staff Education	Limited training and poor retention of protocol knowledge [21]	Interactive, repeated team-based education (workshops, simulations)
Monitoring & Data	Lack of real-time tracking of protocol adherence [11]	Use dashboards, checklists, and order sets for protocol integration and monitoring
Leadership & Culture	Fragmented ownership and weak implementation culture [14]	Strong institutional leadership and promotion of an evidence-based, team-oriented care culture

Comparative studies and meta-analyses

Comparative studies and meta-analyses have been on the rise over the last decade in support of the ERAS protocols as being better than conventional care in pancreatic surgery. Randomized and observational studies all show improvements in recovery timelines, complication rates, and overall safety [46]. In one of the first trials comparing ERAS to standard pathways, the median reduction in LOS was three days, and the overall complications were reduced by 15% in the ERAS group [23]. One of the systematic reviews of over 1,500 patients showed that postoperative problems were less likely when ERAS protocols were used by about 25%, including infections and delayed gastric emptying [47]. The LOS was always reduced by 23 days, and there was no increase in readmission rate or reoperation rates [15].

These results were confirmed by a meta-analysis of 18 studies, including more than 2,800 patients, conducted in 2021, which demonstrated much lower incidences of surgical site infection, pulmonary complications, and clinically relevant pancreatic fistulas in the ERAS group [48]. Nevertheless, pooled estimates were not generalizable due to heterogeneity in the components of the ERAS pathway and the criteria for selecting patients [33]. Importantly, ERAS implementation has not increased perioperative mortality. Conversely, timely detection of the complications due to systematic monitoring could also lead to a reduced rate of serious adverse events [31]. It is important to note that the greatest improvements in outcomes are observed in the settings with high compliance, which are usually characterized by 70 and more percent or the adherence to the ERAS elements [19]. Crucially, ERAS protocols do not compromise oncologic timelines. A number of studies have established that ERAS does not slow down the start of adjuvant chemotherapy; in fact, faster recovery will allow earlier systemic treatment [49]. Collectively, these results support the idea that ERAS does not decrease surgical safety but also improves perioperative recovery compared to standard care.

Economic and quality of life (QoL) implications

Besides the clinical efficacy, ERAS protocols will deliver quantifiable economic and quality-of-life advantages to the patients who undergo a pancreatic surgery. Quantitative research proves the cost-effectiveness of ERAS and its beneficial effect on the short-term well-being of patients [50]. Another prospective study indicated that ERAS saved 3,000-5,000 dollars per patient in pancreatic surgery, mainly due to low complication rates and shorter LOS [12]. There were still such savings even when the training and implementation costs were considered. At the institutional level, ERAS has allowed more consistent recovery patterns and a faster turnover in the operating room, increasing capacity by 812% in certain establishments [18]. Besides the benefit at the system level, ERAS has also been associated with patient satisfaction. Patients often speak of elements of effective pain control, early feeding, and quicker mobilization as improving their experience of recovery [36].

There has also been a better postoperative QoL of ERAS-managed patients. Researchers have used the EORTC QLQ-C30 instrument, which measures physical functioning, emotional well-being, fatigue, and other areas, and they have found the faster recovery of the baseline scores [51]. In a trial, a majority of ERAS patients recovered preoperative QoL in four weeks, as compared to six or more weeks in standard care [17]. In addition to the discharge, ERAS leads to earlier integration into normal life, such as working, and decreased dependency on emergency or readmission [14]. Notably, it does not seem to cause a burden to caregivers, a crucial factor when considering downstream costs and resource utilization [40].

Limitations and future directions

Despite encouraging evidence, several limitations constrain the broad application of ERAS in pancreatic surgery. A major issue is the heterogeneity of ERAS protocols across institutions. Differences in specific elements such as timing of oral intake, use of surgical drains, or criteria for mobilization impede meaningful comparison across studies. Much of the current literature remains retrospective, single-center, and underpowered, reducing external validity. Another critical limitation is the underrepresentation of elderly or frail patients, who are often excluded due to perceived intolerance to aggressive recovery protocols. This omission raises concerns about the generalizability of ERAS benefits to real-world, high-risk populations.

Future research must focus on large, multicenter randomized controlled trials stratified by surgical approach, patient risk profile, and tumor subtype to generate robust, reproducible data. Additionally, long-term monitoring is necessary to evaluate the effects of ERAS on oncologic endpoints such as recurrence-free and overall survival. Emerging technologies, including digital compliance dashboards and AI-assisted pathway optimization, hold promise for personalizing ERAS and improving adherence, as exemplified by pilot programs such as ERAS-CARD. Addressing these knowledge and implementation gaps is critical to optimizing ERAS integration and establishing it as a global standard in pancreatic cancer care.

## Conclusions

ERAS protocols have demonstrated significant benefits in pancreatic cancer resections, including reductions in postoperative morbidity, shorter hospitalizations, improved functional recovery, and timely initiation of adjuvant therapies, without increasing readmissions or mortality. As a comprehensive, evidence-based model involving patient education, nutritional optimization, fluid management, and early mobilization, ERAS aligns well with the goals of modern surgical care. In addition to clinical efficacy, the protocols have shown cost-effectiveness and high levels of patient satisfaction. Nevertheless, implementation remains inconsistent due to variations in institutional capacity, protocol adherence, and care standardization.

This review contributes uniquely by synthesizing clinical outcomes with an in-depth evaluation of operational and structural challenges that influence ERAS effectiveness in pancreatic surgery. It brings attention to underrepresented patient populations, such as the frail and elderly, and emphasizes the importance of protocol fidelity, interdisciplinary coordination, and institutional readiness. Furthermore, it outlines future research priorities, including multicenter trials stratified by surgical and patient variables, and explores the potential of digital tools to enhance compliance and personalization. By bridging current evidence with implementation science, this review provides a strategic framework to optimize ERAS delivery and support its adoption as a standardized component of global pancreatic cancer care.
